# Predictability is attractive: Female preference for behaviourally consistent males but no preference for the level of male aggression in a bi-parental cichlid

**DOI:** 10.1371/journal.pone.0195766

**Published:** 2018-04-10

**Authors:** Ulrike Scherer, Mira Kuhnhardt, Wiebke Schuett

**Affiliations:** 1 Biocentre Grindel, Zoological Institute, Universität Hamburg, Hamburg, Germany; 2 School of Life Sciences, University of Sussex, Falmer, Brighton, United Kingdom; University of Pretoria, SOUTH AFRICA

## Abstract

Although personality traits can largely affect individual fitness we know little about the evolutionary forces generating and maintaining personality variation. Here, we investigated the hypothesis that personality variation in aggression is sexually selected in the monogamous, bi-parental cichlid *Pelvicachromis pulcher*. In this species, breeding pairs form territories and they aggressively defend their territory and offspring against con- and heterospecific intruders. In our mate choice study, we followed up two alternative hypotheses. We either expected females to show a directional preference for a high level and high consistency of aggression (potentially indicating mate choice for male parental quality). Alternatively, we expected females to choose males for (dis-)similarity in the level/consistency of aggression (potentially indicating mate choice for compatibility). Individual level and consistency of aggression were assessed for males and females using mirror tests. After eavesdropping on aggressive behaviour of two males (differing in level and consistency of aggression) females were then allowed to choose between the two males. Males, but not females, showed personality variation in aggression. Further, females generally preferred consistent over inconsistent males independent of their level of aggression. We did not detect a general preference for the level of male aggression. However, we found an above average preference for consistent high-aggression males; whereas female preference for inconsistent high-aggression did not deviate from random choice. Our results suggest behavioural consistency of aggression in male rainbow kribs is selected for via female mate choice. Further, our study underlines the importance of considering both the level and the consistency of a behavioural trait in studies of animal behaviour.

## Introduction

Consistent between-individual differences in behaviour (aka personalities, coping styles or temperaments; [1]) have far-reaching fitness consequences (reviewed in [2, 3, 4]). For example, boldness and aggressiveness have been shown to affect egg fertilization rates [5], survival [6, 7], growth [8, 9], and foraging success [9, 10]. Especially in (bi-) parental species, consistent behavioural differences are thought to heavily affect fitness (reviewed in [1]) because the reproductive success largely depends on parental care behaviour [11, 12]. Parental care behaviour, in turn, is often closely associated with individual personalities [2, 13, 14].

Because personality traits can largely affect individual reproductive success and overall fitness they should likely be considered during mate choice [1, 2, 15, 16]. However, existing studies investigating the link between personalities, or non-sexual behaviour in general, and mate choice are rare and deliver divergent results. Some studies found a general preference for [17–19] or against [20, 21] certain behavioural traits among females of a species. Other studies found females to differ in their mating preference, depending on their own behavioural type, leading to positive assortment [22–24] or dis-assortment [25]. In addition, existing studies on the role of behaviour during mate choice have often neglected the role of between-individual differences in behavioural consistency (but see: [26]), although this is an important personality component that can have diverse fitness implications itself [27–29]. Behavioural consistency in exploration behaviour, for example, is positively correlated with reproductive success in zebra finches, *Taeniopygia guttata*, [26] and consistency in boldness positively correlates with food consumption and collective foraging behaviour in three-spined sticklebacks, *Gasterosteus aculeatus* [30]. Accurate assessment of a potential mate's behaviour (level and consistency) is demanding and costly [31–33] because it requires careful observation. High assessment costs could sometimes outweigh social and reproductive benefits of behavioural consistency promoting also flexible behaviour [29]. Clearly, we need more studies to identify the evolutionary forces shaping the diverse preference pattern, consequently helping us to understand the existence of animal personality variation. Further, a more comprehensive approach is needed including all aspects of behavioural traits (behavioural level and consistency) to fully describe the relation between personality traits and mate choice.

In the present study, we used a correlative approach to investigate the effect of individual aggression (level and consistency) on female mate choice in a bi-parental West African cichlid, the rainbow krib, *Pelvicachromis pulcher*. Breeding pairs of this species raise their offspring in territories and, among other parental duties (e.g. searching for foraging grounds, keeping the brood together), both parents aggressively defend their offspring and territory against any kind of intruders. Therefore, individual differences in aggression are likely to affect reproductive success and should thus be considered during mate choice. We assessed level and consistency of aggression (total number of aggressive behaviours) for all males and females over two mirror tests. Aggressive behaviour directed towards a mirror image reliably reflects aggression towards conspecifics in *P*. *pulcher* [34]. Females were allowed to choose between a high- and a low-aggression male (differing in their consistency) after prior eavesdropping on male aggressive behaviour. If females choose males for their ability to defend offspring and territory (mate choice for male parental quality) we would expect females to generally prefer high- over low-aggression males. Also, several studies found high aggression to be associated with high genetic quality (e.g. [5, 9]). For the behavioural consistency, we expected females to show a general preference for consistent males because this would indicate the reliability of the behaviour allowing a female to predict future parental performance [1, 35]. Also, high behavioural consistency could ease sexual conflict over parental investment through facilitated negotiation over the amount of parental provisioning [35]. Further, high consistency in highly aggressive behaviour can serve as a signal for eavesdropping individuals lowering the number of escalating fights [36]. Alternatively, we expected females to prefer the male being more (dis-) similar to themselves, which could ease synchronisation and/or specialisation of parental abilities and facilitate care coordination (mate choice for compatibility; discussed in [1]).

## Material and methods

### Ethics statement

This work was approved by the German "Behörde für Gesundheit und Verbraucherschutz Hamburg" (permission number 52/16). Stimulus males were used twice in order to reduce the number of animals needed and we used animated conspecifics instead of live conspecifics to reduce stress. Avoiding the risk of injuries during actual fights we determined individual aggressiveness using mirror tests. The number of aggressive behaviours is a good proxy for the probability of fight winning [21, 37] and therefore represents a biologically relevant measure of aggressiveness.

### Fish maintenance

We used laboratory bred rainbow kribs from a breeding stock at the Universität Hamburg, a local supplier (Atlantis Aquarium; Hamburg, Germany; 53°60′58.39″N 10°07′72.39″O) and a wholesaler (Dietzenbach Aquarium GmbH; Dietzenbach, Germany; 50°02′27.32″N 8°80′19.71″O). All fish were maintained in same-sex sibling groups of approx. 20–30 individuals per tank and were fed on 5 days a week with *Artemia* spp.. Holding conditions were standardised using a 12:12 hours light:dark cycle and 100 L fish holding tanks (100 x 50 x 25 cm) containing a layer of sand and plastic aquarium plants. The water (26 ± 1°C water temperature) was internally aerated and filtered and changed once a week. One day prior to the start of the experiment, all fish were measured for their standard length (mean ± SE; males = 4.6 ± 0.1 cm, N = 40; females = 3.8 ± 0.1 cm, N = 39) using ImageJ [38] and transferred to individual housing tanks (25 x 50 x 25 cm, holding conditions as above) for the duration of experimental trials. Each tank was endowed with half a clay pot (8 x 8 x 4 cm) as shelter.

### Experimental outline

We assessed the level and consistency of aggression for all males (N = 40) and females (N = 39) using mirror tests (see *Mirror tests*). Mirror tests were performed twice (5 days in between tests) in order to assess the mean level as well as the degree of individual consistency in aggression and to test for consistent between-individual differences in aggression at population level (repeatability). For mate choice trials, males were paired up to dyads (N = 20) always consisting of two males differing in their level and consistency of aggression, based on their aggression shown during mirror tests (for more details please see *Mirror tests*). Male dyads were used twice during mate choice trials, except one dyad that was only used once. Females were tested for their mate preference once.

Female mate choice trials were conducted in two steps: an observation and a subsequent choice (see *Mate choice trials*). During the observation, females were allowed to eavesdrop on the aggressive behaviour of the two males of a dyad; directed towards their mirror images. Females could then choose between these two males in a dichotomous choice test, a standard procedure suitable to predict mating preferences in cichlids [39, 40]. Several studies have shown that individuals in many fish species gain social information through observing conspecific interactions and later use this information during their own social interactions [18, 21, 41–43].

### Mirror tests

Mirror tests were performed according to Scherer, Buck (34). We started a mirror test by removing filter and heater from an individual's housing tank, and setting up a video camera in front of the tank, one day after introducing fish into their individual housing tanks. After an acclimation of 15 min, a mirror (25 x 50 cm) was introduced on one long side of the tank facing the opening of the clay pot. The focal fish’s behaviour was video-recorded for 12 min. To avoid disturbances, no human was present during recordings and tanks were covered with black plastic foil on three sides. Individuals were tested at the same time of day ± 15 min to avoid potential effects of hunger level or time of day on individual aggression [44, 45] in repeated trials.

Following Scherer, Buck [34], the number of all restrained (frontal displays, left lateral displays, right lateral displays, s-shaped bendings, fast approachings) and overt aggressions (bites) were manually counted from the videos for a duration of 10 min, starting 2 min after the beginning of a video. We calculated the mean aggression level for each individual as the sum of all restrained and overt aggressions (average over both mirror tests). Individual consistency was calculated as behavioural inconsistency: the absolute difference in the number of all aggressive behaviours between the first and second mirror test [30].

Based on the mirror tests, we formed male dyads: the two males within a dyad were matched for size (size difference < 10% of standard length; mean difference ± SE = 2.1 ± 0.3 mm) and family, but were otherwise chosen to have a maximum possible contrast in their aggressive behaviour (mean difference ± SE; level of aggression: 207 ± 28 aggressive behaviours; behavioural inconsistency: 110 ± 18 difference in the number of aggressive behaviours; N = 20 male dyads). Accordingly, males within a dyad were classified into high (mean ± SE = 277 ± 25 aggressive behaviours) and low-aggression males (mean ± SE = 70 ± 15 aggressive behaviours). High- and low-aggression males differed significantly in their mean level of aggression (unpaired Wilcoxon signed-rank test; *W* = 309, *P* < 0.0001; N = 20 male dyads). Likewise, the two males within a dyad were classified into consistent (lower within-individual variation) and inconsistent (higher within-individual variation). Consistent (mean ± SE = 32 ± 8 difference in the number of aggressive behaviours) and inconsistent (mean ± SE = 141 ± 22 difference in the number of aggressive behaviours) males significantly differed in their behavioural inconsistency (unpaired Wilcoxon signed-rank test; *W* = 38, *P* < 0.0001; N = 20 male dyads). For all individuals, the first mirror test was performed before mate choice trials and the second mirror test was performed after mate choice trials. In order to form male dyads used for mate choice trials, we pre-classified males according to their behaviour shown during the first mirror test but final classification was performed a posteriori based on the results of both mirror tests. Differences in the behavioural contrast between the two males of a dyad did not affect female preference. That is, female preference for the preferred male was neither affected by how much the two males of a dyad differed in their mean level of aggression during the mirror tests (linear mixed-effects model; χ^2^_1_ = 1.631, *P* = 0.202; N = 35 mate choice trials, with male pair ID as random effect) nor was it affected by how much the two males of a dyad differed in their behavioural inconsistency (linear mixed-effects model; χ^2^_1_ = 0.281, *P* = 0.596; N = 35 mate choice trials, with male pair ID as random effect).

When considering both male level and consistency classification, our set up resulted in a crossed design with four different male types: consistent high-aggression males, inconsistent high-aggression males, consistent low-aggression males and inconsistent low-aggression males (please see Table 1). Male dyads consisted either of one consistent-high and one inconsistent-low aggression male or, alternatively, they consisted of one inconsistent-high and one consistent-low aggression male.

**Table 1:** Descriptive statistics on male classification of aggression.

	High-aggression males		Low-aggression males	
	Consistent	Inconsistent	Consistent	Inconsistent
N	9	11	11	9
Mean ± SE level	305 ± 43	254 ± 28	42 ± 15	105 ± 22
Mean ± SE inconsistency	44 ± 8	148 ± 33	22 ± 13	133 ± 29

Given are sample sizes and mean ± SE for the level (number of aggressive behaviours) and inconsistency (absolute difference in the number of aggressive behaviours) of aggression within each of the four classifications of male (N_males_ = 40, resulting in N = 20 male dyads) behaviour.

### Mate choice trials

**Fig 1:**
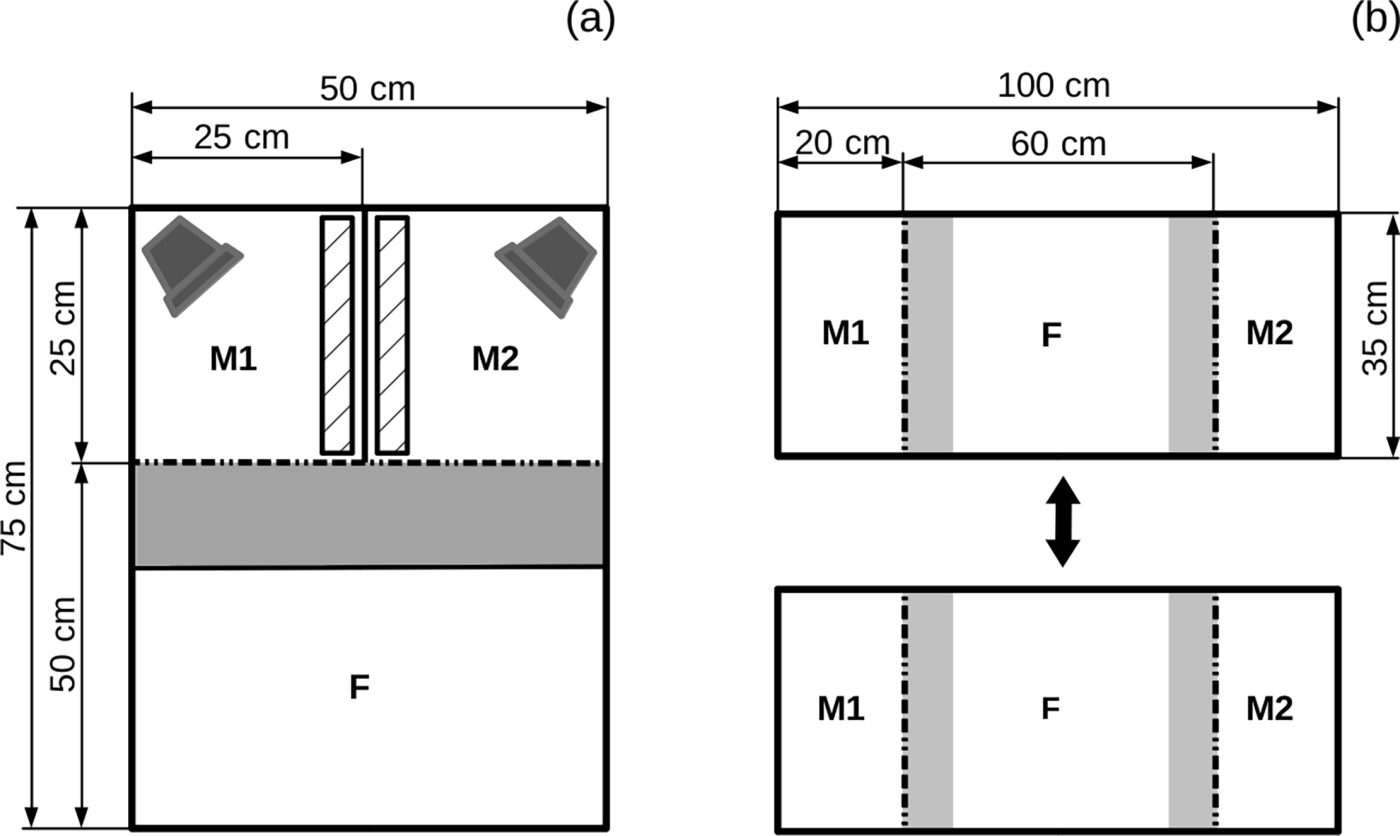
Experimental setup for testing female mating preference. Apparatus for (a) female eavesdropping on male aggression and (b) subsequent female choice. (a) The observation tank (height = 50 cm, water level 10 cm) was divided into three compartments: (F) a female compartment and (M1 and M2) two male compartments. Each male compartment was provided with a half a clay pot (8 x 8 x 4 cm) in a standardised position (objects in dark grey) and a removable mirror (objects with hatching). The female was hidden behind a one-way mirror (slope of 45° to avoid males seeing their mirror image; grey area). (b) The choice chamber (height = 35 cm, water level = 10 cm) was divided into three compartments with the female compartment being in the middle and two male compartments covering the edges of the tank. The female compartment was subdivided into three zones, with the neutral zone being in the middle and the two preference zones for the males on the adjacent sides, each zone alongside the concomitant male compartment (width = 12 cm, refers to approx. two fish lengths; light grey areas). Compartments of observation tank and choice chamber were separated using clear Plexiglas (dashed lines) and/or white Plexiglas (solid lines). Tanks were surrounded with white Plexiglas.

Before each mate choice test, females were allowed to observe male aggressive behaviour. To start the observation, we introduced two males of a dyad into an observation tank (Fig 1A), one male into each of two male compartments. Also, we transferred a randomly chosen female (non-sibling and non-familiar to the males) into the female observer compartment, visually separated from the male compartments using a white partition. After an acclimation period of 15 min, a mirror (25 x 50 cm) was introduced into each male compartment covering the partition between the male compartments (Fig 1A) and the partition visually separating the female compartment from male compartments was removed. Hidden behind a one-way mirror (Fig 1A), the female could observe the two males interacting with their mirrors for 12 min without being seen by males.

Immediately after the observation, the two males and the female observer were transferred to a mate choice chamber (Fig 1B). The two males were randomly assigned to two male compartments and the female was introduced to a female compartment. After an acclimation of 10 min without visual contact, white partitions separating the compartments were gently removed and the first mate choice test period of 12 min started. To take account for a potential side-bias, the trial was repeated immediately after with the males being switched between the male compartments. All fish were again allowed to acclimate for 10 min (under visual separation) before the second test period of 12 min started. During experiments, no observer was present to avoid disturbances. Both test periods were video-recorded from above.

We assessed the association time (female time spent in a male's preference zone (Fig 1B), sum of both test periods, sec) for each male from the videos using Ethovision XT 11 (Noldus, Wageningen, The Netherlands). Videos were analysed for 10 min, starting 2 min after the beginning of a video. Female mating preference was then quantified from both test periods as the strength of preference for each male: the association time for one male was divided by the association for both males (e.g. [25, 46, 47]). Further, we calculated female side bias as the time a female spent in one preference zone relative to the amount of time spent in both preference zones (sum of both test periods). A female was considered side-biased when she spent more than 80% of the test time in just one preference zone [25, 48, 49]. We decided a priori to exclude side-biased preference data from the analysis (N = 1 mate choice trial) (e.g. [25, 50]). Another three mate choice trials were excluded because of damaged video files, resulting in final N = 35 trials used for preference analyses (including N = 18 male dyads).

### Statistical analysis

All statistical analyses were performed in R version 3.4.0 [51]. Repeatability of aggressive behaviour was calculated for males and females separately using linear mixed effects models (LMMs) implemented in the *rptR*-package [52]. Repeatability calculations were performed with 1000 bootstrapping runs and 1000 permutations. Significant repeatability was given when the 95% confidence interval (CI) did not include 0. Further, we tested for a sex difference in the mean level and inconsistency of male and female aggression (N_total_ = 79, consisting of N_males_ = 40 and N_females_ = 39) fitting two linear models (LMs): one model was fit on the level of aggression and the other model was fit on the behavioural inconsistency, both models contained the sex as predictor variable.

We tested for a general preference for high- over low-aggression males using an LMM (lme4-package, [53]) with female preference for high-aggression males (including both consistent and inconsistent high-aggression males; N_trials_ = 35) as response variable. We included male dyad ID as random effect but otherwise did not include any fixed effects (aka null model). Deviation from random choice would be revealed if the 95% CI of the mean does not include 0.5. Further, we tested for a preference for consistent over inconsistent males (including both low- and high-aggression; N_trials_ = 35) using the same approach: we ran a null model with female preference for consistent males as response and included male dyad ID as random effect.

In order to test the possibility that female preference for the consistency and the level of male aggression are interdependent we further assessed deviation from random choice of female preference for consistent high-aggression (N_trials_ = 17) and consistent low-aggression (N_trials_ = 18) using the above method. To avoid redundancy, we did not analyse the remaining behavioural combinations (inconsistent high-aggression and inconsistent low-aggression) in the same way. Due to our experimental design, female preference for consistent high-aggression males were directly inverse to female preference for inconsistent low-aggression males. Likewise, female preference for inconsistent high-aggression males and for consistent low-aggression males were directly inverse.

Also, we tested for a difference in female preference between consistent high- and consistent low-aggression males. We fit an LMM on female preference for consistent males (N_trials_ = 35) including male behavioural type combination (consistent high-aggression and consistent low-aggression) as fixed effect and male dyad ID as random effect. Similarly, we tested for a difference in female preference between consistent and inconsistent high-aggression males (N_trials_ = 35) fitting an LMM on female preference for high-aggression males, again, including male behavioural type combination (consistent high-aggression and inconsistent high-aggression) as fixed effect and male dyad ID as random effect. For all models, we calculated effect sizes (partial R^2^) for fixed effects following Nakagawa and Schielzeth [54] using the *r2glmm*-package [55]. For non-significant fixed effects we report the partial R^2^ deriving from the model before the term was dropped. Model assumptions were visually assured using model diagnosis plots. For all analyses, female strength of preference was *arcsine-square root*-transformed for normality of the residuals.

The prediction that females might show a preference for behavioural (dis-) similarity in aggression is based on the assumption that females show personality variation for aggressiveness. However, overall female aggression was not repeatable (please see Results). Therefore, we cannot present a reliable measure of female aggressive behaviour. Thus, we only present an analysis of female preference for (dis-) similarity in the supplement (S1 File).

We provide our raw data including behavioural data of mirror test (S2 File) and behavioural data obtained during mate choice trials (S3 File). Also, we supply our R code used for preference analyses (S4 File).

## Results

**Fig 2:**
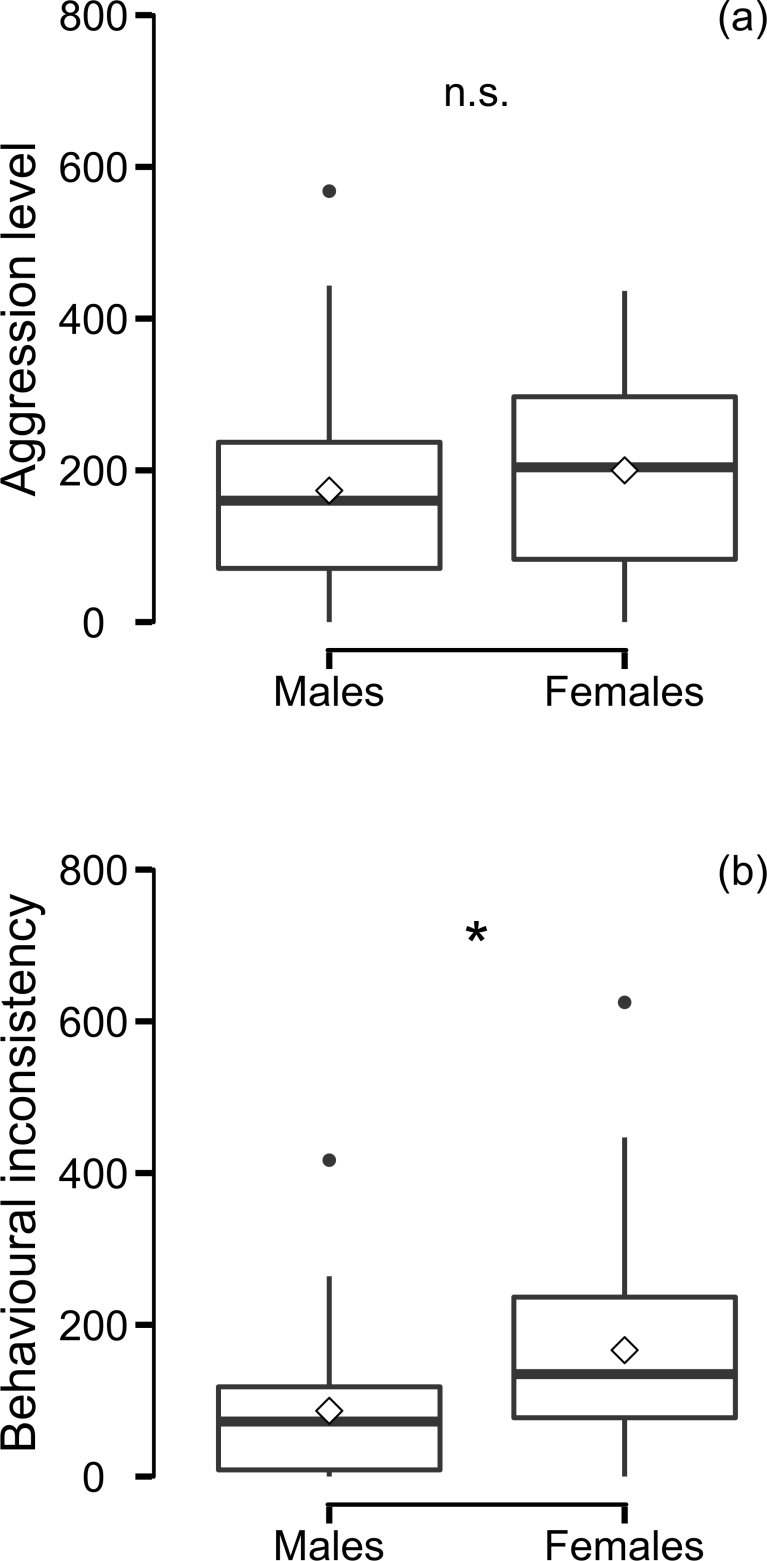
**Comparison of male and female (a) level and (b)** inconsistency **of aggression.** Level (number of aggressive behaviours) and inconsistency (absolute difference in the number of aggressive behaviours) of male and female aggressive behaviour. Boxplots present original data with mean (◊), mean (-) and 1.5 interquartile ranges, significance indicated (significant: *; non-significant: n.s.).

Male aggressive behaviour (mean ± SE = 166 ± 15 aggressive behaviours) was significantly repeatable (R ± SE = 0.660 ± 0.092, CI = [0.452, 0.807]; N_trials_ = 80, N_males_ = 40). In contrast, female aggressive behaviour (mean ± SE = 196 ± 17 aggressive behaviours) was not repeatable (R ± SE = 0.176 ± 0.135, CI = [0.000, 0.460]; N_trials_ = 78, N_females_ = 39). Males (mean ± SE = 170 ± 22 aggressive behaviours) tended to be less aggressive than females (mean ± SE = 219 ± 17 aggressive behaviours) (LM; estimate ± SE = -62 ± 35, F_1,77_ = 3.110, P = 0.082; R^2^ = 0.039, CI = [0.160, 0.100]; N_males+females_ = 79 males and females; Fig 2A). Further, females (mean ± SE = 0.252 ± 0.032) were less consistent than males (mean ± SE = 0.143 ± 0.024) (LM; estimate ± SE = 80 ± 26, F_1,77_ = 9.581, P = 0.003; R^2^ = 0.111, CI = [0.016, 0.263]; N_males+females_ = 79; Fig 2B).

**Fig 3:**
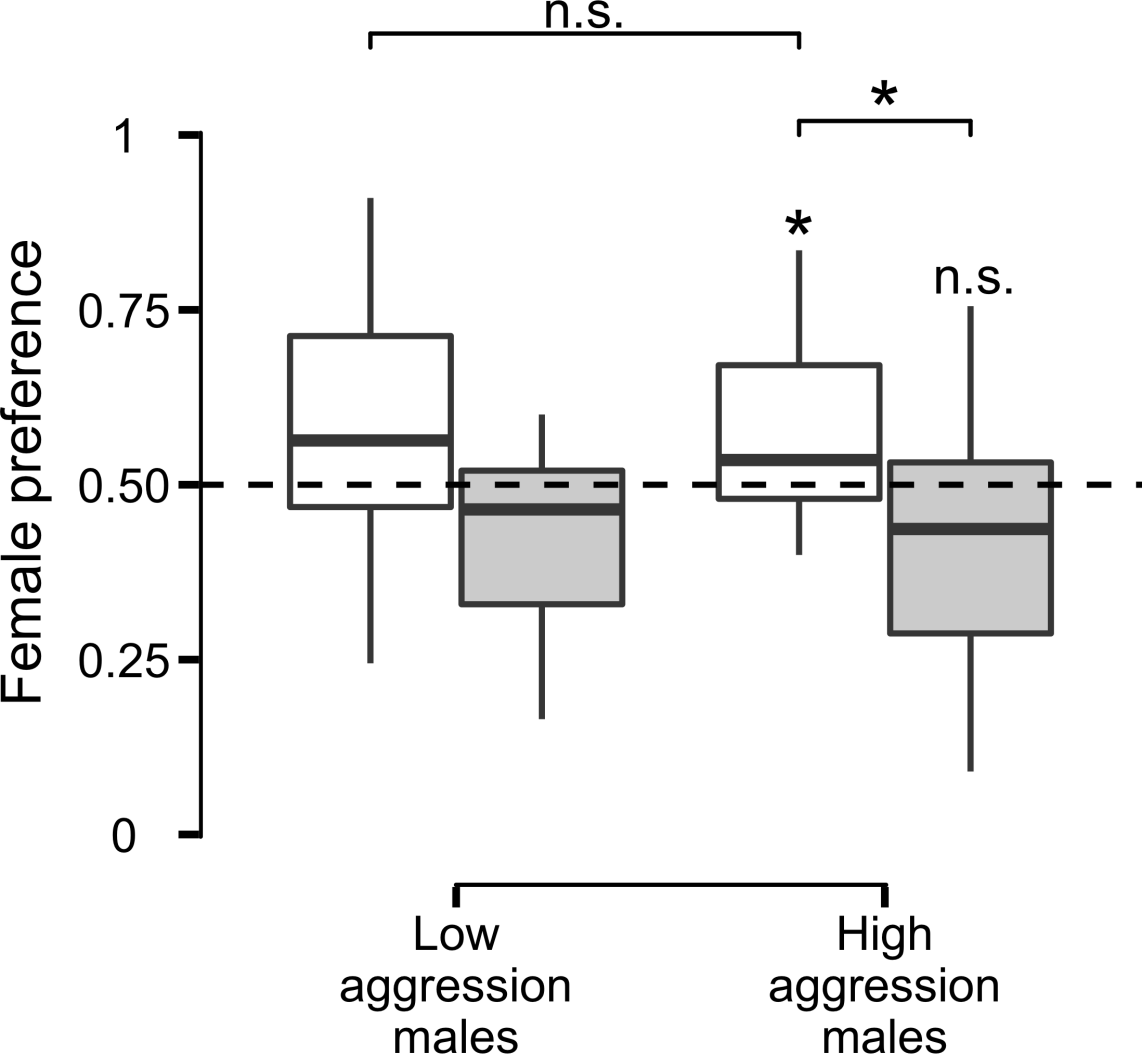
Female preference. Deviation from random choice (female preference = 0.50; dashed line) for high- and low-aggression males, split into consistent (white filling) and inconsistent (grey filling). Boxplots present original data with median (-) and 1.5 interquartile ranges, significance indicated (significant: *; non-significant: n.s.). Please note, female preference for consistent high-aggression and inconsistent low-aggression males are directly inverse, as well as inconsistent high-aggression and consistent-low preference scores.

Females did not show a general preference for high- over low-aggression males (including consistent and inconsistent males; mean preference = 0.492; CI = [0.416, 0.567]; N = 35). In contrast, females generally preferred consistent over inconsistent males (including high- and low-aggression males; mean preference = 0.577; CI = [0.511, 0.641]; N = 35). Female preference for consistent males was mainly driven by a significant above average preference for consistent high-aggression males (mean preference = 0.571; CI = [0.508, 0.633]; Fig 3) whereas female preference for consistent low-aggression males did not deviate from random choice (mean preference = 0.584; CI = [0.459, 0.704]; Fig 3). However, preference scores for consistent-high and consistent low-aggression males did not statistically differ from each other (LMM; estimate ± SE = 0.014 ± 0.064, χ^2^ = 0.054, P = 0.816; R^2^ = 0.002, CI = [0.000, 0.148]; N = 35; Fig 3). Female preference for consistent high-aggression males was significantly higher than female preference for inconsistent high-aggression males (LMM; estimate ± SE = -0.154 ± 0.064, χ^2^ = 5.057, P = 0.025; R^2^ = 0.191, CI = [0.022, 0.441]; N = 35; Fig 3). Inconsistent low-aggression males were the least preferred (Fig 3).

## Discussion

We found that only male but not female *P*. *pulcher* showed personality variation in aggressiveness. Males and females did not differ in their level of aggression but males showed significantly higher behavioural consistency. Further, females did not show a general preference for high- over low-aggression males. Instead, female preference for the level of male aggression was dependent on the consistency in which male aggression was expressed; that means consistent high-aggression males received significant above average preference scores but inconsistent high-aggression males did not. Finally, females generally preferred consistent over inconsistent males no matter whether these males were classified as high- or low-aggression.

The sex difference in the consistency of aggression might indicate there are different selective regimes acting on male and female behavioural consistency. Although both rainbow krib parents engage in offspring and territory defence a typical division of labour can be observed with males usually doing a greater proportion of territory defence and females providing more direct offspring care. Such a division of labour with specific sex roles during parental care can be commonly observed in cichlid fish species (e.g. [56, 57, 58]). Due to this parental role allocation, the selective pressure on consistent aggression might be higher for male than for female *P*. *pulcher*. In other words, while females may benefit from choosing consistent high-aggression males in terms of better offspring and territory defence, there may not be such benefits for consistent female aggression. Instead, selection may actively favour flexibility of female aggression due to possibly high costs of consistent aggression in close proximity to the offspring [59, 60]; i.e. during direct offspring care female aggression could easily be misdirected towards offspring when expressed consistently. In a closely related sister species, the convict cichlid, *Amatitlania siquia*, behavioural flexibility increased reproductive success of behaviourally mismatched breeding pairs through behavioural convergence [29]. In the present study, female inconsistency might potentially allow them to flexibly adjust their behaviour to the needs of that very moment.

Because both sexes in the rainbow krib provide parental care, we expected mate choice for behavioural compatibility to be likely. But females did not prefer behaviourally (dis-) similar males (see Supplement), which may be attributed to the fact that we did not find personality variation for female aggression. Instead, females showed a preference for consistent high-aggression males suggesting mate choice for (parental) quality. A high level of aggression could ensure a male's ability to defend offspring and territory, while behavioural consistency could signal a female how reliable the information is. High consistency in aggressiveness could allow a female to predict male future parental care performance (in defence behaviour) from male aggression shown prior to reproduction [1, 35, 36]. Also, females could benefit from choosing consistent high-aggression males if these males provide genetic benefits for the offspring. For instance, aggressiveness has been shown to correlate with food intake and growth (reviewed in [61]), reproductive success (reviewed in [62]) and fat storage (in zebra finches, [62]). Notably, we did not set up a choice condition testing female preference for consistent high-aggression vs. consistent low-aggression. Hence, we cannot conclusively prove a directional female preference for the level of male aggression.

We found male behavioural consistency to affect female mate choice although females were allowed to observe male aggressive behaviour only once. Possibly, the behavioural consistency within one trial correlates with the behavioural consistency between repeated behavioural measurements. That is, an individual that behaves homogenously at one time (e.g. number of aggressive behaviours evenly distributed throughout the observation) might possibly also behave homogenously throughout time (thus showing a similar number of aggressive behaviours anytime later). On the contrary, an individual that behaves very heterogeneously within one observation (e.g. high fluctuation in the frequency of performing aggressive behaviours) might show higher heterogeneity between observations. Such a transition from within- to between-observational consistency would allow a female to predict future (parental) behaviour from just one observation. Due to our correlative experimental design, female preference could also be related to consistency in a different behaviour (e.g. general swimming behaviour, activity), or even to a non-behavioural trait (e.g. colouration) that might be correlated to behavioural consistency in aggressiveness. Further examinations using experimental manipulations of the natural behaviour are inevitable to disentangle the behaviour from other, possibly correlated traits.

The strength of our study lies within the consideration of both personality compounds: level and consistency of aggression. In animal personality research, large attempts have been made to understand the evolution of individual differences in the level of behaviour. However, the effects of individual differences in the behavioural consistency have mostly been unattended (but see: [25, 26, 30]). Our study shows the effects of the behavioural level can be tightly linked to the consistency in which the behaviour is expressed. We highlight the importance of considering both aspects of a personality trait (the level and the consistency) and encourage future research to use a more holistic experimental design in studies on animal personality. Clearly, the power of our experimental design is limited by a lack of male behavioural data from the observation phase of mate choice trials. Although males were behaviourally consistent on population level, individuals differed in their degree of consistency. While the behaviour of "consistent" males should confidently match their classification we cannot be conclusively sure that the behavioural level shown by "inconsistent" males during the observation matched their classification. The uncertainty in "inconsistent" male aggression might have interfered with our testing of female preference for male aggression level and could have weakened the signal. However, the classification "inconsistent" does not necessarily mean a male shows high behavioural instability. Instead, it solely means that individual's behavioural consistency is lower compared to the consistency of the other male within one male dyad. Generally, the behavioural consistency of all males, including "consistent" and "inconsistent" was relatively high.

## Conclusion

In summary, we found males and females to be equally aggressive but females were less consistent in their aggressiveness, which might be attributed to the parental roles during offspring care leading to sexual selection favouring consistent male aggression (advantage in offspring and territory defence) and disfavouring consistent female aggression (dangerous for offspring). Female preference for consistent high-aggression males might indicate female choice for parental care quality or male genetic quality. However, in order to determine, which of these two non-exclusive evolutionary mechanisms (mate choice for parental care or mate choice for intrinsic quality) is relevant follow-up breeding experiments disentangling direct behavioural from genetic benefits are necessary. Females generally preferred high consistency though a high level of aggression was only preferred in combination with high consistency. This might indicate that the behavioural consistency (indicating the quality of the signal) is more important than the behavioural level. However, the adaptive benefit of behavioural consistency (independent of the behavioural level) remains to be tested in our target species. This would be especially worthwhile with regard to parental performance as behavioural consistency is expected to provide reproductive benefits associated with the predictability of behaviour: facilitation of parental role specialisation and/or eased negotiation over amount of offspring care [1, 35]. Our results highlight that behavioural consistency is an essential component of personality traits that should not be overlooked in the behavioural sciences.

## Supporting information

S1 File Analysis of female preference for behavioural (dis-) similarity. (DOCX)

S2 File Raw data obtained during mirror tests. (XLSX)

S3 File Raw data obtained during mate choice trials. (XLSX)

S4 File R code for preference analyses. (R)
