# Supplementary material for: Predictability is attractive: Female preference for behaviourally consistent males but no preference for the level of male aggression in a bi-parental cichlid
Source: PLoS One. 2018 Apr 10;13(4):e0195766. doi: 10.1371/journal.pone.0195766 (PMC5892930; doi:10.1371/journal.pone.0195766)
Supplement: S1 File — (DOCX) [file pone.0195766.s001.docx]

**Predictability is attractive: Female preference for behaviourally consistent males but no preference for the level of male aggression**

**Ulrike Scherer * ^1^, Mira Kuhnhardt ^1^ & Wiebke Schuett ^1,2^**

^1^ Biocentre Grindel, Zoological Institute, Universität Hamburg, Hamburg, Germany

^2^ School of Life Sciences, University of Sussex, Falmer, Brighton, United Kingdom

* Corresponding author

E-mail: u.k.scherer@gmail.com

**S1 File. Analysis of female preference for behavioural (dis-) similarity**

**Statistical analysis**

Data analysis was performed in R version 3.4.0 (1). To test for an effect of male-female behavioural (dis-) similarity on female preference (please see main text for the assessment of female preference) we fit an LMM (linear mixed-effects model) on female preference for high-aggression males (consistent and inconsistent) (N = 35). The model included relative similarity in the behavioural level and relative similarity in the behavioural consistency as fixed effects; male ID was included as random effect. Relative similarity (for level and consistency, respectively) was calculated as the female's similarity with the low-aggression/inconsistent male (absolute value of the difference) minus the female's similarity with the high aggression/consistent male (absolute value of the difference) (2). Thus, positive values indicate the female is behaviourally more similar to the high aggression/consistent male than to the low-aggression/inconsistent male and vice versa. Before analysis, predictor variables were z-transformed for standardization using the *GenABEL*-package (3). For modeling, we used the *lme4*-package (4). The minimum adequate model was fit using a backward model selection approach. Effect sizes (partial R^2^) with CIs were calculated for fixed effects following Nakagawa and Schielzeth (5) using the *r2glmm*-package (6). For insignificant fixed effects, R^2^ and CIs of the model before the term was dropped were reported. Raw data used for this analysis are provided in S3 File. Our R script for running the preference analysis is presented in S4 File.

**Results**

Female preference for high aggression males was neither affected by relative similarity in the behavioural level (LMM; standardized estimate ± SE = -0.011 ± 0.027, χ^2^_1_ = 0.142, *P* = 0.707; R^2^ = 0.004, CI = [0.000, 0.153]; N = 35) nor was it affected by relative similarity in consistency (LMM; standardized estimate ± SE = -0.031 ± 0.030, χ^2^_1_ = 0.954, *P* = 0.329; R^2^ = 0.028, CI = [0.000, 0.222]; N = 35).

**References**

1. R Core Team. R: A language and environment for statistical computing. Vienna, Austria: R Foundation for Statistical Computing; 2017.

2. Scherer U, Kuhnhardt M, Schuett W. Different or alike? Female rainbow kribs choose males of similar consistency and dissimilar level of boldness. Animal Behaviour. 2017;128:117-24.

3. GenABEL project developers. GenABEL: genome-wide SNP association analysis. R package version 1.8-0. ; 2013.

4. Bates D, Mächler M, Bolker B, Walker S. Fitting linear mixed-effects models using lme4. Journal of Statistical Software. 2015;67(1):1-48.

5. Nakagawa S, Schielzeth H. A general and simple method for obtaining R^2^ from generalized linear mixed-effects models. Methods in Ecology and Evolution. 2013;4(2):133-42.

6. Jaeger B. R2glmm: computes R squared for mixed (multilevel) models. R package version 0.1.1. 2016.
